# A Rare Urothelial Malignant Transformation in a Mature Cystic Teratoma of the Ovary

**DOI:** 10.7759/cureus.17285

**Published:** 2021-08-18

**Authors:** Moito Iijima, Iori Kisu, Tetsuro Shiraishi, Rie Irie, Nobumaru Hirao

**Affiliations:** 1 Department of Obstetrics and Gynecology, Federation of National Public Service Personnel Mutual Aid Associations Tachikawa Hospital, Tokyo, JPN; 2 Department of Obstetrics and Gynecology, Keio University School of Medicine, Tokyo, JPN; 3 Department of Pathology, Federation of National Public Service Personnel Mutual Aid Associations Tachikawa Hospital, Tokyo, JPN

**Keywords:** malignant transformation, mature cystic teratoma, urothelial carcinoma, ovarian cancer, pathological examination

## Abstract

Approximately 0.17-2% of mature cystic teratomas undergo malignant transformation, of which squamous cell carcinoma (SCC) is the most common, accounting for 80% of these cases. Urothelial malignant transformation is extremely rare. The present study involves a 58-year-old patient who visited the hospital with discomfort in the lower abdomen. USG and pelvic MRI showed a left ovarian mature cystic teratoma. Left salpingo-oophorectomy was performed, and pathological examination revealed urothelial carcinoma transformation of the mature cystic teratoma morphologically and immunohistochemically. No metastasis to other organs was identified by CT after the surgery. Additional surgery, including total hysterectomy, right salpingo-oophorectomy, omentectomy, and dissection of pelvic and para-aortic lymph nodes, was performed without complications. No tumors were identified elsewhere, and the patient's stage was confirmed as IA. She had an uneventful postoperative course and was discharged 10 days later. CT showed no metastasis or recurrence six months later.

## Introduction

Mature cystic teratoma (MCT) of the ovary is the most common type of ovarian germ cell neoplasm, occurring in approximately 20% of all ovarian neoplasms [1, 2]. The incidence of malignant transformation in MCTs is 0.17-2% [3]. More than 80% of MCT malignant transformations are squamous cell carcinomas (SCCs) arising from the ectoderm, and most of the remaining are carcinoid tumors or adenocarcinomas [4]. Urothelial carcinomas arising from an MCT are extremely rare and very few have been reported in the literature [4-11]. Here, we present the case of a woman with an incidental finding of a urothelial carcinoma arising from a left ovarian MCT, who underwent successful surgical treatment. We also briefly review the pertinent literature.

## Case presentation

**Figure 1:**
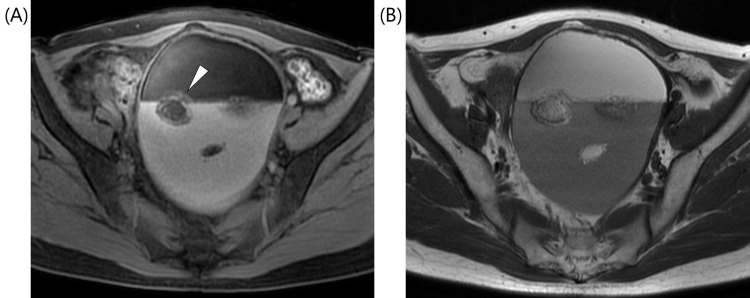
Preoperative MRI findings. (A) On axial T1-weighted image (T1WI), the fluid stored in the ventral side of the cyst showed a high signal. Hairball-like floating material was seen inside the cyst (white arrowhead). (B) In fat-suppressed T1WI, the fluid stored in the ventral side of the cyst showed a low signal.

A 58-year-old postmenopausal woman, gravida 1, para 1, presented at our hospital, with complaints of lower abdominal discomfort. Her initial pelvic examination revealed a soft mass in the pelvic cavity. Transvaginal USG revealed a 15-cm cystic mass, with a smooth surface and a fatty component, within the left ovary. Pelvic MRI revealed that the ventral side of the left ovarian cyst showed high signal intensity on T1-weighted images (T1WIs) and low signal intensity on fat-suppressed T1WIs, suggesting an accumulation of fluid with a fatty component and hairball-like floating material inside. These imaging results were indicative of MCT (Figures 1A-1B).

As none of the mass was found to be solid, malignancy was not suggested. The tumor markers were as follows: SCC antigen, 3.6 ng/mL (normal, <1.5 ng/mL); carcinoembryonic antigen (CEA), 1.3 ng/mL (normal, <5.0 ng/mL); carbohydrate antigen (CA)-125, 7.7 IU/mL (normal, <35.0 IU/mL); and CA19-9, 72.0 IU/mL (normal, <37.0 IU/mL). Other laboratory tests revealed no abnormalities.

The patient underwent a left salpingo-oophorectomy via open abdominal surgery. Gross examination of the left ovary revealed a smooth surface cyst, which was 16 cm in diameter. The right ovary and uterus were grossly normal. Tumor rupture was not observed during surgery. On the cut surface, the cyst was filled with yellowish, creamy fluid and hairs, which are classic features of MCT.

**Figure 2:**
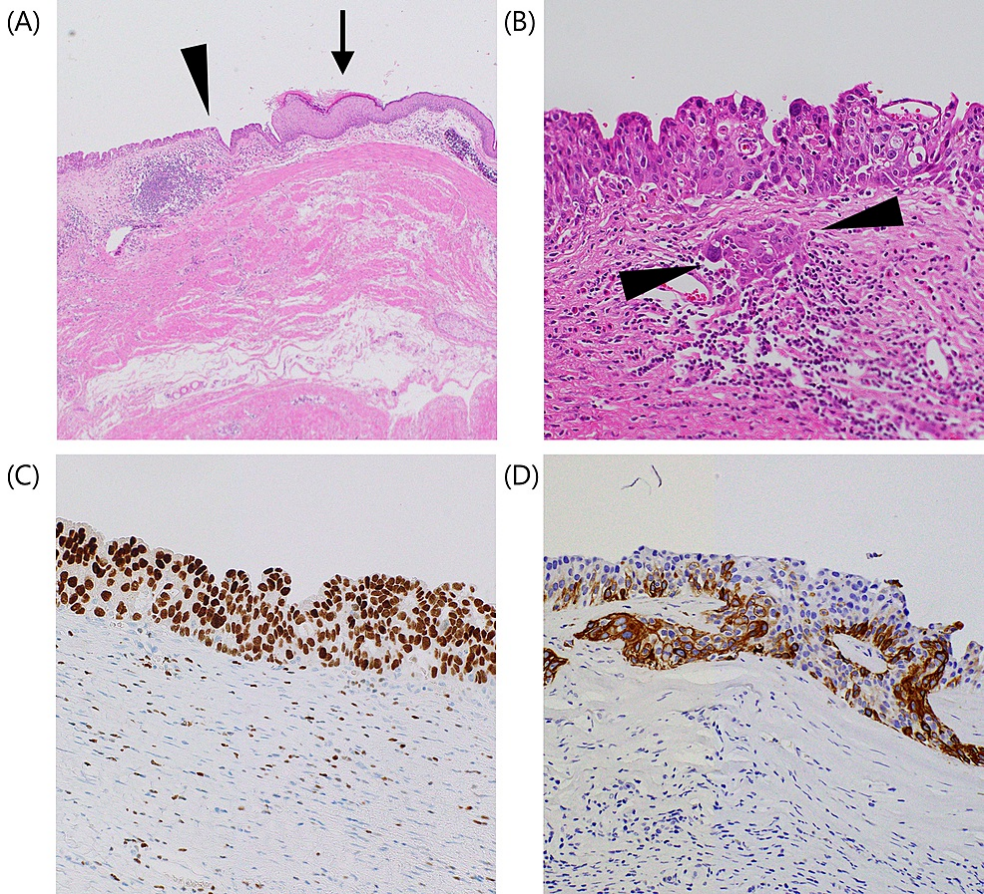
Pathological findings of the urothelial malignant transformation. (A) Low power view of the epithelium covering the inner surface of the cyst in the left ovary consisting of areas of the urothelium (black arrowhead) and squamous epithelium (black arrow) (H&E 4X magnification). (B) High power view of urothelial carcinoma with black arrowheads pointing at nests of invasive malignant epithelial cells in ovarian stroma (H&E 20X magnification). (C) High power view of tumor cells showing the nuclei, which were strongly positive for GATA3 (Immunohistochemistry (IHC) 40X magnification). (D) High power view of tumor cells showing the cytoplasm, which was positive for cytokeratin (CK) 5/6 (IHC 40X magnification).

Microscopically, the left ovary showed various mature tissues, including skin appendages, glandular epithelium covering the bronchi, and fatty tissue. The epithelium covering the inner surface of the cyst had areas of urothelium and squamous epithelium (Figure 2A).

Atypical epithelial cells were found only in the area of the uroepithelium, and grew as papillary-like structures, while the urothelium had nuclear pleomorphism, hyperchromatism, and increased mitotic activity, and there was evidence of microinvasion of nests of malignant epithelial cells into the ovarian stroma (Figure 2B). Immunohistochemistry (IHC) analysis showed that the nuclei of atypical cells were strongly positive for GATA3 (Figure 2C) and the cytoplasm of atypical cells was positive for cytokeratin (CK) 5/6 (Figure 2D). The final pathology report confirmed the existence of urothelial carcinoma in an ovarian MCT. Cytological analysis of the intraoperatively collected ascites was negative.

Postoperative CT revealed no metastasis to other organs. The patient underwent a secondary operation, which included a total abdominal hysterectomy, right salpingo-oophorectomy, omentectomy, and dissection of pelvic and para-aortic lymph nodes, without complications. Pathologically, no tumors were identified in other organs, and therefore, the patient was diagnosed as stage IA (International Federation of Gynecology and Obstetrics 2014). She had an uneventful postoperative stay and was discharged 10 days later, without sequelae. At her six-month follow-up, CT did not show distant metastasis or local recurrence, and no elevation of SCC or CA19-9 was observed.

## Discussion

MCT is one of the most common ovarian tumors, accounting for approximately 20% [1]. Malignant transformation occurs in 0.17-2% of MCTs, and is rarely recognized preoperatively [4, 12, 13]. Of these malignant transformations, urothelial carcinomas arising from MCT are extremely rare, and only eight such cases have been reported in the literature to date [4-11]. Urothelial carcinoma of the ovary has been described as a primary ovarian carcinoma with definite urothelial features but no benign, metaplastic, and/or proliferating Brenner tumor identified [14]. The diagnosis of primary invasive urothelial carcinoma in the present case was made by morphology and immunohistochemical analysis, which demonstrated GATA3 in the malignant cells.

An overview of the salient features of the previously mentioned eight cases, as well as the present patient, is presented in Table 1 [4-11].

**Table 1:** Reported cases of urothelial carcinoma arising from mature cystic teratoma of the ovary. ATH: Abdominal total hysterectomy; BPLA: Bilateral pelvic lymphadenectomy; BSO: Bilateral salpingo-oophorectomy; CA: Carbohydrate antigen; FIGO: International Federation of Obstetrics and Gynecology; LSO: Left salpingo-oophorectomy; NA: Not available; NED: No evidence of disease; OMT: Omentectomy; RSO: Right salpingo-oophorectomy.

	Study	Age (years)	Menopause	Symptoms	Tumor size (cm)	Laterality	Tumor marker elevated	FIGO stage	Primary surgery	Further treatment	Follow-up
1	Lee HH et al. (1999) [8]	67	Post	Voiding difficulty, lower abdominal pain	14 x 7 x 5	Left	CA-125 CA19-9	IC	ATH + BSO + OMT	Chemotherapy; carboplatin, etoposide	NED 5 months
2	Kido A et al. (1999) [4]	48	NA	NA	NA	NA	NA	IC	NA	NA	NDA
3	Yamaguchi K et al. (2007) [11]	48	NA	Abdominal mass	20	Left	CA-125 CEA SCC	IC	ATH + BSO + OMT		NED 10 years
4	Al-Rayyan ES et al. (2009) [5]	45	Pre	Pain, bleeding	8	Left	NA	IA	LSO	Surgical staging	NED >5 years
5	Lee OJ (2010) [9]	52	Pre	Abdominal mass	22 x 19 x 5	Right	NA	IA	RSO	NA	NED 15 months
6	Chuang HY et al. (2015) [6]	54	Post	Abdominal mass	20 x 13 x 21	Right	CA-125 CA19-9	IA	ATH + BSO + OMT + BPLA	None	NED 8 months
7	Dasgupta S et al. (2015) [7]	50	Post	Pain, abdominal swelling	11 x 9 x 6	Right	CA-125	IA	ATH + BSO	None	NED
8	Mathew MR et al. (2018) [10]	50	NA	Pain	12 x 8 x 5	Right	-	IA	ATH + BSO	None	NED 3 months
9	Present case	58	Post	Discomfort in the lower abdomen	16	Left	CA19-9 SCC	IA	LSO	Surgical staging	NED 5 months

The mean age of the patients from all nine cases was 52 years. Of the patients for which data were available, four were postmenopausal and only two were premenopausal, which is compatible with previous reports on malignant transformation of MCTs [15]. In most cases, the tumor was more than 10 cm in diameter and the patients were diagnosed at an early stage, such as stage IA or IC, requiring surgery, including salpingo-oophorectomy. In most cases, mural nodules were noted on MRI or CT imaging performed prior to the primary surgery, although they were not found in the present case. Among the eight reports, except for the present one, there is no report using IHC with GATA3 for the diagnosis of urothelial carcinoma. Only one patient underwent adjuvant chemotherapy, which is included etoposide and carboplatin [8]. In all cases for which data were available, except in the present case, CA125 levels were elevated.

Some studies have reported various risk factors for the malignant transformation of MCT. Age greater than 45 years, preoperative tumor size >9.9 cm, and elevated levels of serum tumor markers (CA125, CA19-9, CEA, and SCC) were noted as being risk factors [5, 12, 13]. Of the tumor markers, SCC and CA125 have been reported to indicate high sensitivity, specificity, and diagnostic efficiency in the preoperative diagnosis of MCT with malignant transformation to squamous cell carcinoma [16].

GATA3 is a dual-zinc finger transcription factor that regulates gene expression in many developing tissues, which is positive in 76% of urothelial carcinomas [17]. It can be effectively used to diagnose urothelial carcinoma, but it has also been reported to be positive in other tumors, including breast cancer. This marker can also be effectively used in predicting the probable grade and invasion in biopsy material with poor morphological characteristics [17].

The pathogenesis of malignant transformation of MCTs is not yet completely known. Iwasa A et al. [18] suggested that squamous cell carcinoma in MCT may originate from metaplastic squamous epithelium, rather than teratomatous squamous epithelium. Lee OJ et al. [9] reported a case in which components of normal urothelial epithelium and urothelial carcinoma were adjacent to each other, suggesting that prolonged and persistent stimulation of teratomatous or metaplastic urothelial epithelium by some irritating material within the teratoma may cause urothelial carcinoma in MCT.

MRI is useful in the preoperative diagnosis of the malignant transformation of MCT, but sometimes no indication of malignant transformation is noted, as in the present case. Most mural nodules in MCTs without malignant transformation are called Rokitansky protuberances. However, because MCTs are composed of various components of the tritubular lobe, it has been reported that even without malignant transformation, they form mural nodules that differ from the Rokitansky protuberances, which are composed of neural tissue, thyroid tissue, and fibrous stroma [19]. In the absence of fat or calcification indicating teeth or bone within the mural nodule, it is difficult to rule out malignant transformation using MRI. Diffusion-weighted images, which show high signal intensities in the presence of malignancy, may also show areas of high signal intensity in MCT without malignant transformation, making differentiation by MRI alone difficult.

When it is difficult to differentiate malignant transformation using MRI, F-fluoro-2-deoxy-d-glucose (FDG) positron emission tomography/CT (PET/CT) may be considered. It has been reported that MCT with malignant transformation can be differentiated from MCT without malignant transformation by FDG-PET/CT, as the former has higher FDG accumulation when using the maximum standardized uptake value (SUVmax) 2.9 as a cutoff value [20].

## Conclusions

Urothelial carcinoma arising from MCT is extremely rare. The conditions for considering the possibility of urothelial malignant transformation may be the same as those for other malignant transformations; if the size is large, if there is a transmural soft tissue component inside the teratoma, if the patient is older than 45 years, or if tumor markers are high, the possibility of malignant transformation, including that of the urothelium, should be considered. FDG-PET and MRI may both be considered for the diagnosis of malignant transformation of MCT.
